# Evaluating the mosquito vector range for two orthobunyaviruses: Oya virus and Ebinur Lake virus

**DOI:** 10.1186/s13071-024-06295-5

**Published:** 2024-05-07

**Authors:** Siyuan Liu, Xiaoyu Wang, Fei Wang, Wahid Zaman, Cihan Yang, Doudou Huang, Haixia Ma, Jinglin Wang, Qiyong Liu, Zhiming Yuan, Han Xia

**Affiliations:** 1 Key Laboratory of Virology and Biosafety, Wuhan Institute of Virology, Chinese Academy of Sciences, Wuhan, Hubei China; 2 University of Chinese Academy of Sciences, Beijing, China; 3 Yunnan Tropical and Subtropical Animal Virus Disease Laboratory, Yunnan Animal Science and Veterinary Institute, Kunming, China; 4 National Key Laboratory of Intelligent Tracking and Forecasting for Infectious Diseases, National Institute for Communicable Disease Control and Prevention, Chinese Center for Disease Control and Prevention, Beijing, China; 5 Hubei Jiangxia Laboratory, Wuhan, China

**Keywords:** Mosquito, Vector, Orthobunyavirus, Oya virus, Ebinur Lake virus

## Abstract

**Background:**

Mosquito-borne viruses cause various infectious diseases in humans and animals. Oya virus (OYAV) and Ebinur Lake virus (EBIV), belonging to the genus *Orthobunyavirus* within the family *Peribunyaviridae*, are recognized as neglected viruses with the potential to pose threats to animal or public health. The evaluation of vector competence is essential for predicting the arbovirus transmission risk.

**Methods:**

To investigate the range of mosquito vectors for OYAV (strain SZC50) and EBIV (strain Cu20-XJ), the susceptibility of four mosquito species (*Culex pipiens pallens*, *Cx. quinquefasciatus*, *Aedes albopictus*, and *Ae. aegypti*) was measured through artificial oral infection. Then, mosquito species with a high infection rate (IR) were chosen to further evaluate the dissemination rate (DR), transmission rate (TR), and transmission efficiency. The viral RNA in each mosquito sample was determined by RT-qPCR.

**Results:**

The results revealed that for OYAV, *Cx. pipiens pallens* had the highest IR (up to 40.0%) among the four species, but the DR and TR were 4.8% and 0.0%, respectively. For EBIV, *Cx. pipiens pallens* and *Cx. quinquefasciatus* had higher IR compared to *Ae. albopictus* (1.7%). However, the EBIV RNA and infectious virus were detected in *Cx. pipiens pallens*, with a TR of up to 15.4% and a transmission efficiency of 3.3%.

**Conclusions:**

The findings indicate that *Cx. pipiens pallens* was susceptible to OYAV but had an extremely low risk of transmitting the virus. *Culex pipiens pallens* and *Cx. quinquefasciatus* were susceptible to EBIV, and *Cx. pipiens pallens* had a higher transmission risk to EBIV than *Cx. quinquefasciatus*.

**Graphical Abstract:**

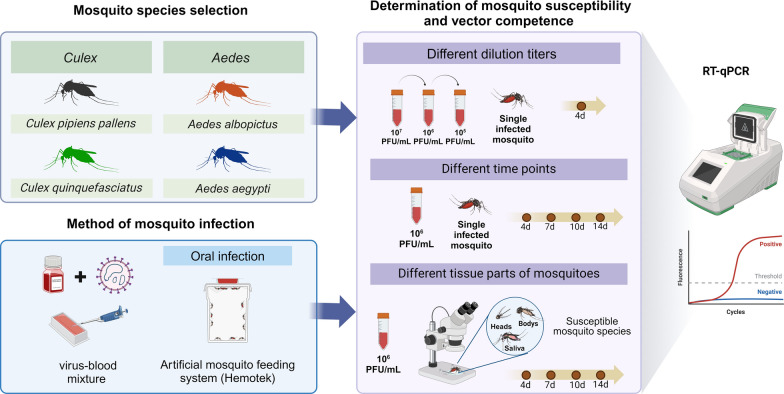

**Supplementary Information:**

The online version contains supplementary material available at 10.1186/s13071-024-06295-5.

## Background

Mosquitoes are important vectors, carrying and transmitting a wide range of pathogens, including arboviruses primarily from the families *Peribunyaviridae*, *Togaviridae*, *Flaviviridae*, *Phenuiviridae*, and *Reoviridae* [1], as well as various types of parasites [2].

The family *Peribunyaviridae*, genus *Orthobunyavirus*, contains three-segmented, negative-stranded RNA viruses transmitted exclusively by arthropods [3]. So far, nearly 200 viruses of the genus *Orthobunyavirus* have been identified, and they are grouped into 20 serogroups [4]. Multiple viruses belonging to the Simbu and Bunyawera serogroups have been found in various mosquito species, including *Culex* spp., *Aedes* spp., and *Anopheles* spp. [5–7]. Orthobunyaviruses such as Oropouche virus (OROV) and Bunyamwera virus (BUNV) can cause fever or encephalitis in humans [8, 9], while Schmallenberg virus (SBV) and Akabane virus (AKAV) infect livestock [10, 11].

Oya virus (OYAV) and Ebinur Lake virus (EBIV) belong to the *Orthobunyavirus* genus [4]. Oya virus and Cat Que virus (CQV) are classified under the same virus species, *Orthobunyavirus catqueense* [12]. The OYAV was initially isolated from a pig suspected to be infected with the Nipah virus in Malaysia in 2000 [13]. Subsequent studies have detected or isolated OYAV from invertebrate or vertebrate hosts in China, Vietnam, and India [14–19]. Ebinur Lake virus, a member of the Bunyamwera serogroup, was initially isolated from the pools of *Culex modestus* in Xinjiang Province, China, and was later detected in various samples from Russia (mosquitoes, ticks, and wild birds) and Kenya (*Dasymys incomtus* and *Herpestes ichneumon*) [20, 21]. Experimental investigations have demonstrated that these viruses efficiently infect and replicate within various cell lines derived from vertebrates and mosquitoes [18, 22]. Furthermore, studies have proven the lethality of OYAV in suckling and C57BL/6 adult mice [18] and the fatality of EBIV in BALB/c adult mice [23]. Despite the lack of confirmed cases of OYAV and EBIV, a serological survey in Malaysia revealed that 93% of pigs within pig farming zones tested positive for OYAV [13], and the seroprevalence of OYAV in pigs in Yunnan, China, exceeded 30% [18]. Moreover, antibodies against EBIV have previously been detected in residents of Xinjiang, with an IgM-positive rate of 8% and an IgG-positive rate of 12% in the 211 human serum samples [22]. These findings underscore the global threat that OYAV and EBIV pose to animal or public health, necessitating further research.

Two of the more prevalent mosquito genera that transmit arbovirus in China are *Aedes* and *Culex* [7]. *Culex pipiens pallens* and *Cx. quinquefasciatus*, both members of the *Culex pipiens* complex, are predominantly found in the northern and southern regions of China, respectively [24]. The *Cx. pipiens* complex plays a crucial role in transmitting arboviruses, particularly the West Nile virus [25, 26]. *Aedes albopictus* and *Ae. aegypti* are recognized as the primary vectors of DENV [27, 28]. *Aedes albopictus* is a widespread species in the southern regions of China [29]. In China, *Ae. aegypti* is distributed in the tropical cities of Hainan, Guangxi, and Guangdong Province, but the geographical distribution has spread to Yunnan with the changes in the climate [30, 31]. Our previous work demonstrated that EBIV can spread into saliva in *Ae. aegypti* after artificial oral infection, which poses a potential transmission risk for EBIV by mosquito [32]. Meanwhile, the susceptibility of *Culex* and *Ae. aegypti* mosquitoes to CQV has been reported from India [33]. Nevertheless, there is a need for more relevant mosquito studies on EBIV and OYAV isolates in China, where different genotypes/isolates of the virus, mosquito species, and geographical strains may impact vector competence [34, 35].

The goal of this study was to investigate the vector competence of four mosquito species, *Cx. pipiens pallens*, *Cx. quinquefasciatus*, *Ae. albopictus*, and *Ae. aegypti*, to OYAV and EBIV through artificial oral infection. This research will enhance our understanding of the transmission risk for these neglected orthobunyaviruses.

## Methods

### Mosquito species and rearing

This study used four mosquito species, *Cx. pipiens pallens* (Beijing strain, kindly provided by China CDC), *Cx. quinquefasciatus* (Wuhan strain), *Ae. albopictus* (Jiangsu strain, kindly provided by China CDC), and *Ae*. *aegypti* (Rockefeller strain, kindly provided by Qian Han from Hainan University). The feeding methods and processes were the same as described earlier [32].

### Cell lines and viral stock

Golden hamster kidney cells (BHK, RRID CVCL_1914) and pig kidney cells (PK 15, RRID CVCL_2160) were cultured in Dulbecco’s modified Eagle’s medium (Gibco, Grand Island, NY, USA) containing 10% FBS and 1% penicillin/streptomycin (Gibco) at 37 ℃ and 5% CO_2_.

Oya virus (strain SZC50), isolated from midges in Yunnan, China [18], and EBIV (strain Cu20-XJ), isolated from *Cx. modestus* in Xinjiang, China, were used in this study [20]. The titers of OYAV and EBIV were determined using the plaque formation assay, and their working stocks were found to be 1.4 × 10^7^ plaque-forming units ml^−1^ (PFU/ml) and 7.0 × 10^7^ PFU/ml, respectively.

### Adult mosquito infection through artificial oral infection

For infections through an artificial mosquito feeding system (Hemotek, Blackburn, UK), 3- to 5-day-old adult females were starved for 12 to 24 h and fed with an infected blood meal (a mixture of the defibrinate horse blood and virus supernatant). The titer of the virus-blood mixture was determined at the same time by plaque assay. The detailed procedures for oral infection can be referred to in our previously published article [36]. Since our group described the results of EBIV infection in *Ae. aegypti* previously [32], we did not infect *Ae. aegypti* with EBIV in this study anymore.

To establish the appropriate concentration of OYAV and EBIV in different mosquito species, *Cx. pipiens pallens*, *Cx. quinquefasciatus*, *Ae. albopictus*, and *Ae. aegypti* exposed to different virus-blood concentrations (OYAV: two serial titers ranging from 10^6^–10^5^ PFU/ml; EBIV: three serial titers ranging from 10^7^–10^5^ PFU/ml) were subsequently collected at 4 days post-exposure (dpe) for viral RNA determination. Then, the mosquitoes were exposed to OYAV and EBIV at 10^6^ PFU/ml, according to the result in the previous step, and the exposed mosquitoes were collected at 4, 7, 10, and 14 dpe to determine viral RNA by qRT-PCR.

Furthermore, the virally susceptible mosquito species were used to determine the dissemination rate, transmission rate, and transmission efficiency. The *Cx. pipiens pallens* (OYAV) and *Cx. pipiens pallens/Cx. quinquefasciatus* (EBIV) were exposed via artificial oral infection (final viral titer of 10^6^ PFU/ml), and viral RNA in the bodies, heads, and saliva of the exposed mosquitoes at 4, 7, 10, and 14 dpe was detected.

All mosquito individuals, tissues, and saliva were stored in 250 μl Roswell Park Memorial Institute (RPMI) 1640 medium (Gibco, Grand Island, USA) supplemented with 2% penicillin/streptomycin/gentamicin solution (Gibco, Grand Island, USA) and kept at − 80 °C until use. The vector competence of the mosquitoes was evaluated by calculating the infection rate (IR; number of positive bodies/the total number of mosquitoes tested), dissemination rate (DR; number of positive heads/the number of positive bodies), transmission rate (TR; number of positive saliva/the number of positive bodies), and transmission efficiency (number of infected saliva/the total number of mosquitoes tested) [32, 37].

### Evaluation of viral RNA by RT-qPCR

All samples were initially homogenized using a Low Temperature Tissue Homogenizer Grinding Machine (Servicebio, Wuhan, China) (operating frequency = 60 Hz, operation time = 10 s, pause time = 10 s, cycles = 3, and setting temperature = 4 °C), followed by centrifugation for 5 min at 10,000×*g* and 4 °C. The total RNA of each sample was extracted using 200 μl of the prepared samples with an automated nucleic acid extraction system, following the manufacturer’s instructions (NanoMagBio, Wuhan, China).

Using the CFX96 Touch Real-Time PCR Detection System (Bio-Rad, California, USA), viral RNA copies of each sample were quantified. For OYAV, the Luna® Universal Probe One-Step RT-qPCR Kit (NEB, Ipswich, USA) was used, and for EBIV, the HiScript II One Step qRT-PCR Probe Kit (Vazyme, Nanjing, China) was used. The primer and probe sets used are presented in previous studies [19]. The cutoff for positive samples determined via RT-qPCR was Ct (OYAV) < 36 and Ct (EBIV) < 35. The positive cutoff value was evaluated by comparing paired serial ten-fold dilutions either inoculated on cells or assayed via RT-qPCR (Additional file 1: Table S1) [38]. The equation for the standard curve is shown in Additional file 2: Table S2 and used to calculate the viral genome copies in each sample. Some positive mosquitoes (Ct = 22–29) were homogenized and inoculated into BHK-21 cells, and the CPE showed after 2 days post-infection (dpi) (Additional file 3: Fig. S1A), which indicated not only viral RNA but also live viruses were present in the mosquito samples.

### Statistical analysis

GraphPad Prism 8.0.2 (GraphPad Software Inc) was used to analyze all the data. Fisher’s exact test was used to compare the mosquito’s infection, dissemination, and transmission rates between different treatments. One-way ANOVA with Tukey’s multiple comparison was used to compare the mean of the genome copies among more than two data sets. *P* ≤ 0.05 was considered statistically significant.

The graphical abstract was created with BioRender.com (https://www.biorender.com/), and authorization for publication had been granted.

## Results

### The susceptibility of adult *Culex* and *Aedes* mosquitoes to different titers of OYAV and EBIV

**Fig. 1:**
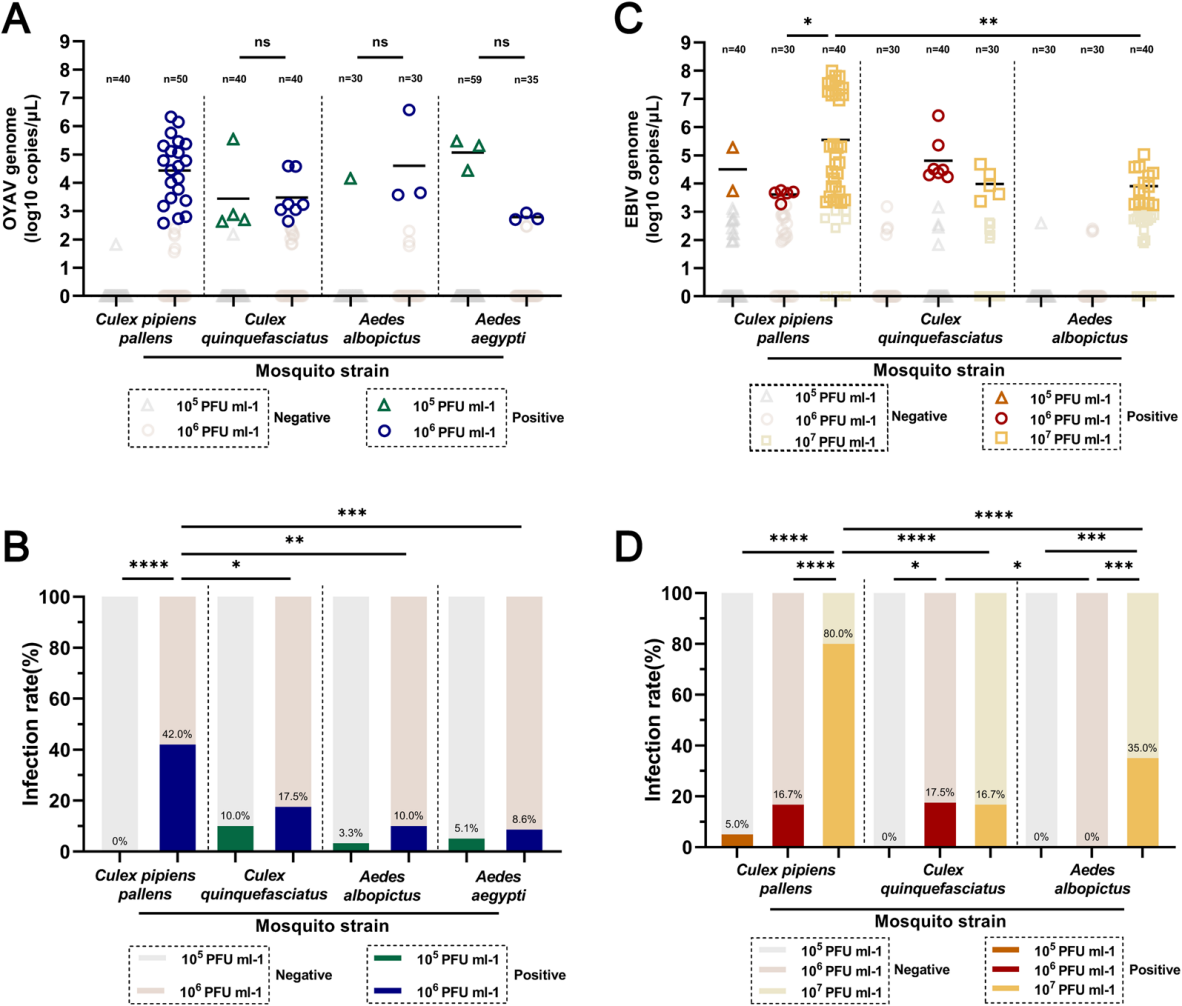
Infection rates for four mosquito species exposed to different doses of OYAV or EBIV through artificial oral infection. Viral RNA copies (**A**) and infection rates (**B**) of *Culex pipiens pallens*, *Cx. quinquefasciatus*, *Aedes albopictus*, and *Ae. aegypti* at 4 days after feeding on blood meals with continuous dilution of OYAV from 10^6^ to 10^5^ PFU/ml. Viral RNA copies (**C**) and infection rates (**D**) of *Cx. pipiens pallens*, *Cx. quinquefasciatus*, and *Ae. albopictus* were evaluated after 4 days of feeding on blood meals with a continuous dilution of EBIV from 10^7^ to 10^5^ PFU/ml. Each dot represents an individual mosquito, and the gray dots indicate samples with Ct values > 36 (OYAV) and > 35 (EBIV). Infection rates were analyzed with Fisher’s exact test, and mean viral RNA copies/μl were analyzed with one-way ANOVA with Tukey’s multiple comparison (*P ≤ 0.05, **P ≤ 0.01, ***P ≤ 0.005, ****P ≤ 0.0001). The “ns” indicates that no significant difference was observed

The infectivity of mosquitoes consuming artificial blood meals with varying virus titers was investigated. Four mosquito species—*Cx. pipiens pallens*, *Cx. quinquefasciatus*, *Ae. albopictus*, and *Ae. aegypti*—were fed blood meals containing 10^5^ and 10^6^ PFU/ml of OYAV, respectively. The infection rates and viral RNA loads were assessed at 4 dpe (Fig. 1A, B). No OYAV-positive mosquitoes were detected in *Cx. pipiens pallens* among the 40 tested mosquitoes when using a titer of 10^5^ PFU/ml. However, when using 10^6^ PFU/ml OYAV, the IR significantly increased (*P* < 0.0001) and reached 42.0% among 50 tested mosquitoes, with an average viral RNA concentration of 10^4.4^ copies/μl and a peak of 10^6.3^ copies/μl. For *Cx. quinquefasciatus*, *Ae. albopictus*, and *Ae. aegypti*, IR was not significantly affected by the titers for OYAV, with IR remaining < 18% at higher titers. Meanwhile, at the exposure titer of 10^6^ PFU/ml for OYAV, the IR in *Cx. pipiens pallens* was significantly higher than in *Cx. quinquefasciatus*, *Ae. albopictus*, and *Ae. aegypti* (*P* = 0.0211; 0.0025; 0.0006, respectively). There was no significant difference in mean viral RNA copies among exposed *Cx. quinquefasciatus*, *Ae. albopictus*, and *Ae. aegypti* at varying exposure titers.

Three mosquito species—*Cx. pipiens pallens*, *Cx. quinquefasciatus*, and *Ae. albopictus*—were exposed to EBIV in blood meals with titers ranging from 10^5^, 10^6^, and 10^7^ PFU/ml, respectively. Their IR and viral replication were assessed at 4 dpe (Fig. 1C, D). In *Cx. pipiens pallens,* a positive correlation was observed between blood meal titers and IR, with an IR of 80.0% at the highest titer of 10^7^ PFU/ml, significantly surpassing the rates at lower titers (all *P* < 0.0001). The mean viral RNA copies in positive mosquitoes were 10^5.6^ copies/μl, peaking at 10^8.0^ copies/μl, notably higher than in lower titer infections. For *Cx. quinquefasciatus*, EBIV RNA was not detected at a blood meal titer of 10^5^ PFU/ml. However, at 10^6^ PFU/ml and 10^7^ PFU/ml, the IR increased to 17.5% and 16.7%, respectively, with mean viral RNA copies of 10^4.8^ copies/µl and 10^4.0^ copies/µl. In *Ae. albopictus*, there were no infections at 10^5^ PFU/ml or 10^6^ PFU/ml. Nevertheless, at a 10^7^ PFU/ml titer, the IR rose to 35.0%, with mean viral RNA copies of 10^3.9^ copies/μl and a maximum of 10^5.0^ copies/μl. At the highest titer of 10^7^ PFU/ml, *Cx. pipiens pallens* exhibited a significantly higher IR than other mosquito species (*P* < 0.0001). At a titer of 10^6^ PFU/ml, *Cx. pipiens pallens* and *Cx. quinquefasciatus* displayed similar IRs, both higher than that of *Ae. albopictus* (*P* = 0.0173).

### Dynamic variations in viral titers in adult mosquitoes exposed to OYAV or EBIV via artificial oral infection

**Fig. 2:**
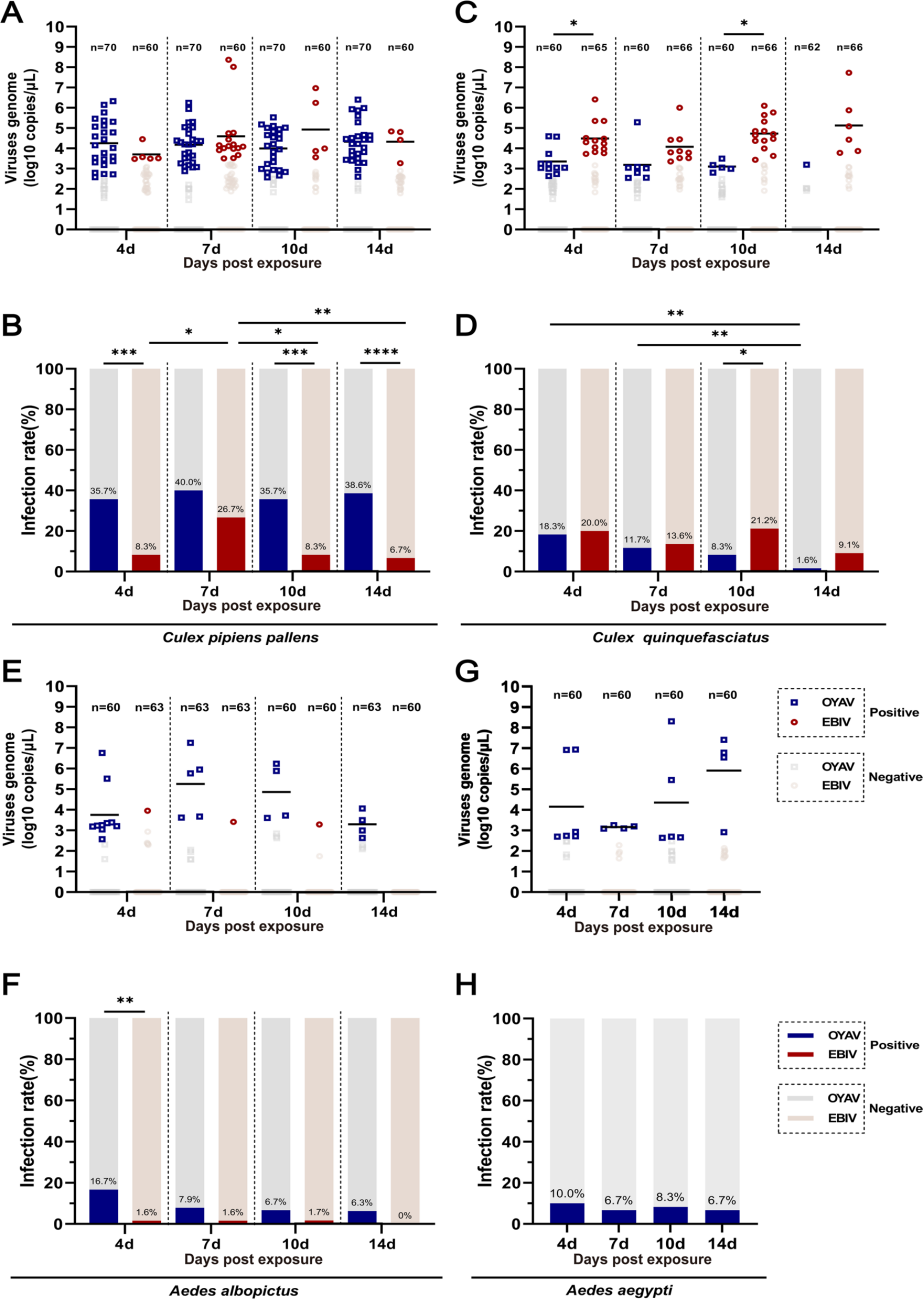
Infection rates for four mosquito species exposed to OYAV or EBIV at 10^6^ PFU/ml through oral feeding. Viral RNA copies in *Culex pipiens pallens* (**A**), *Cx. quinquefasciatus* (**C**), *Aedes albopictus* (**E**), and *Ae. aegypti* (**G**) and infection rates in *Cx. pipiens pallens* (**B**), *Cx. quinquefasciatus* (**D**), *Ae. albopictus* (**F**), and *Ae. aegypti* (**H**) at 4, 7, 10, and 14 days after feeding on a blood meal containing 10^6^ PFU/ml OYAV and EBIV. Each dot represents an individual mosquito, and the gray dots indicate samples with Ct values > 36 (OYAV) and > 35 (EBIV). The infection rates were analyzed with Fisher’s exact test, and mean viral RNA copies/μl were analyzed with one-way ANOVA with Tukey’s multiple comparison (*P ≤ 0.05, **P ≤ 0.01, ***P ≤ 0.005, ****P ≤ 0.0001)

To assess adult mosquitoes’ susceptibility to OYAV and EBIV infections, they were exposed through artificial oral feeding at an average titer of 3.8 × 10^6^ PFU/ml, with samples collected at 4, 7, 10, and 14 dpe for RNA analysis (Fig. 2A–H).

For *Cx. pipiens pallens* (Fig. 2A, B), the OYAV IR remained relatively stable from 4 to 14 dpe, fluctuating between 35.7 and 40.0%. The mean viral RNA copies in positive mosquitoes varied slightly, ranging from 10^4.0^ to 10^4.3^ copies/μl. In contrast, EBIV infection resulted in significantly lower IRs at 4 (*P* = 0.0003), 10 (*P* = 0.0003), and 14 (*P* < 0.0001) dpe compared to OYAV. The peak IR for EBIV was observed at 7 dpe (26.7%), notably higher than the rates at 4, 10 (both *P* = 0.0148), and 14 dpe (*P* = 0.0060). The mean viral RNA copies in EBIV-positive mosquitoes were 10^4.6^ copies/μl, with some exceeding 10^8^ copies/μl.

For *Cx. quinquefasciatus* (Fig. 2C, D), the OYAV IR decreased from 18.3% at 4 dpe to 1.6% at 14 dpe. The mean viral RNA copies remained relatively constant, with a maximum of 10^3.4^ copies/μl at 4 dpe. Ebinur Lake virus infection results in higher IRs at 4 and 10 dpe (20.0% and 21.2%, respectively) than those at 7 and 14 dpe (13.6% and 9.1%, respectively). The mean viral RNA copies were highest at 14 dpe, with one positive mosquito having a viral RNA copy of 10^7.7^ copies/μl and a mean of 10^5.1^ copies/μl. The mean viral RNA copies of EBIV-positive mosquitoes at 4 and 10 dpe were significantly higher than those exposed to OYAV (*P* = 0.0366 and 0.0120, respectively).

For *Ae. albopictus* (Fig. 2E, F), the IR decreased over time following OYAV infection, with the highest IR of 16.7% at 4 dpe and the lowest at 6.3% at 14 dpe. At 7 dpe, positive mosquitoes' mean viral RNA copies were 10^5.3^ copies/μl, with one mosquito having viral RNA copies of 10^7.3^ copies/μl. EBIV showed low infectivity in *Ae. albopictus*, with only one positive detection at 4, 7, and 10 dpe (IR of 1.6%, 1.6%, and 1.7%, respectively), whereas no positive detections were achieved among 60 mosquitoes at 14 dpe.

For *Ae. aegypti* (Fig. 2G, H), the OYAV IR did not significantly change from 4 to 14 days, remaining between 6.7% and 10%. The mean viral RNA copies in positive mosquitoes also showed no significant variation, with the highest mean viral RNA copies observed at 14 dpe (10^5.9^ copies/μl). Some mosquitoes exhibited higher viral RNA copies, reaching 10^8.3^ copies/μl.

### Dissemination and transmission dynamics of OYAV and EBIV in mosquitoes

**Fig. 3:**
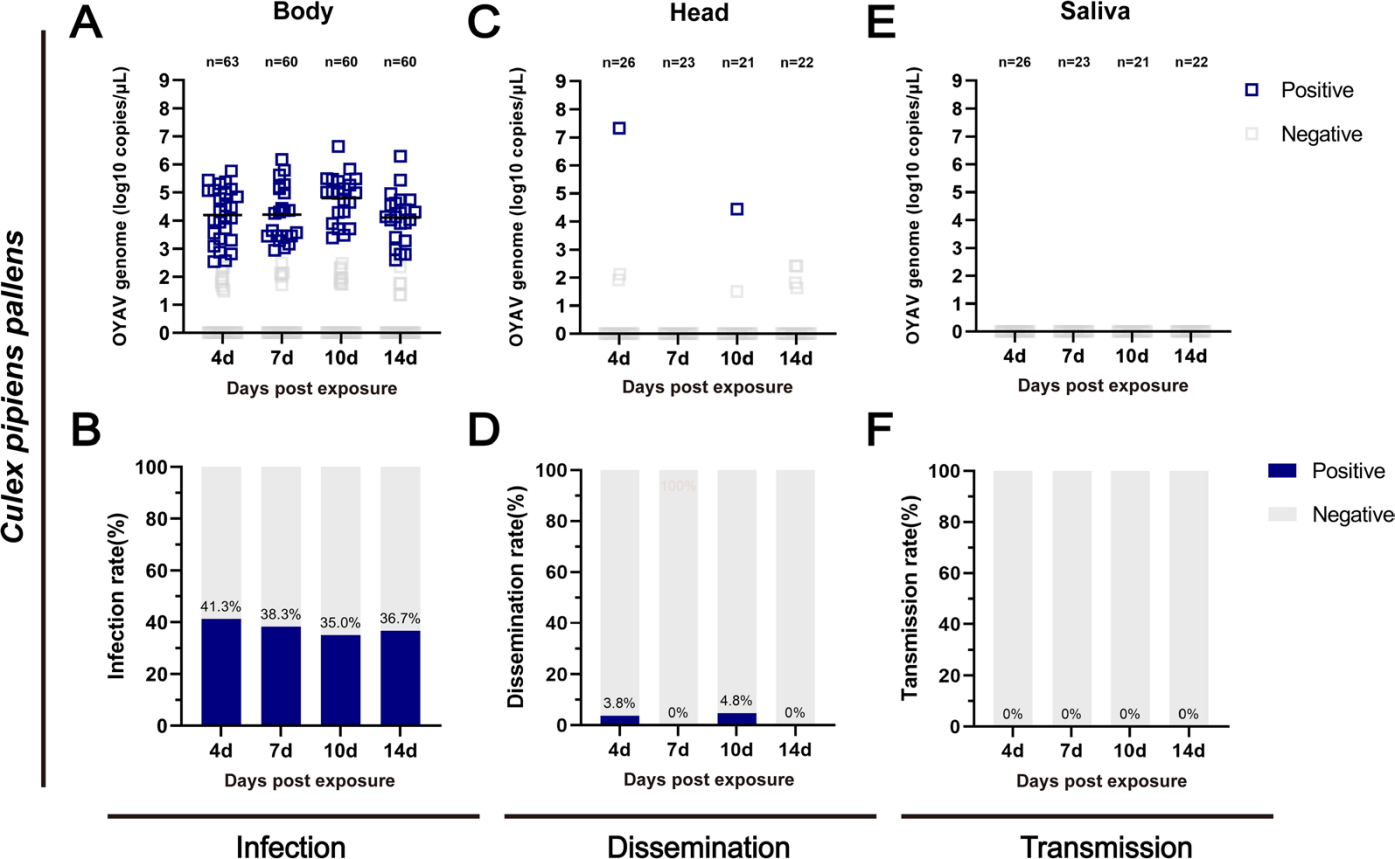
OYAV infection, dissemination and transmission in *Culex pipiens pallens*. Viral RNA copies in the bodies (**A**), heads (**C**), and saliva (**E**) samples and infection rates (**B**), dissemination rates (**D**), and transmission rates (**F**) of mosquitoes at 4, 7, 10, and 14 days after feeding on a blood meal containing 3.2 × 10^6^ PFU/ml OYAV. Each dot represents an individual mosquito, and the gray dots indicate samples with a Ct value > 36. The rates were analyzed with Fisher’s exact test, and mean viral RNA copies/μl were analyzed with one-way ANOVA with Tukey’s multiple comparison (*P ≤ 0.05, **P ≤ 0.01, ***P ≤ 0.005, ****P ≤ 0.0001)

Based on the infection rate in the above result, * Cx. pipiens pallens* was selected to explore the dissemination and transmission dynamics of OYAV. Only one positive head (10^7.3^ copies/μl) was detected at 4 dpe among 26 body-positive mosquitoes and one (10^4.5^ copies/μl) at 10 dpe among 21 samples, corresponding to the low DR of 3.8% and 4.8% (Fig. 3A–D). In addition, no OYAV-positive saliva sample was detected (Fig. 3E, F) in *Cx. pipiens pallens*.

**Fig. 4:**
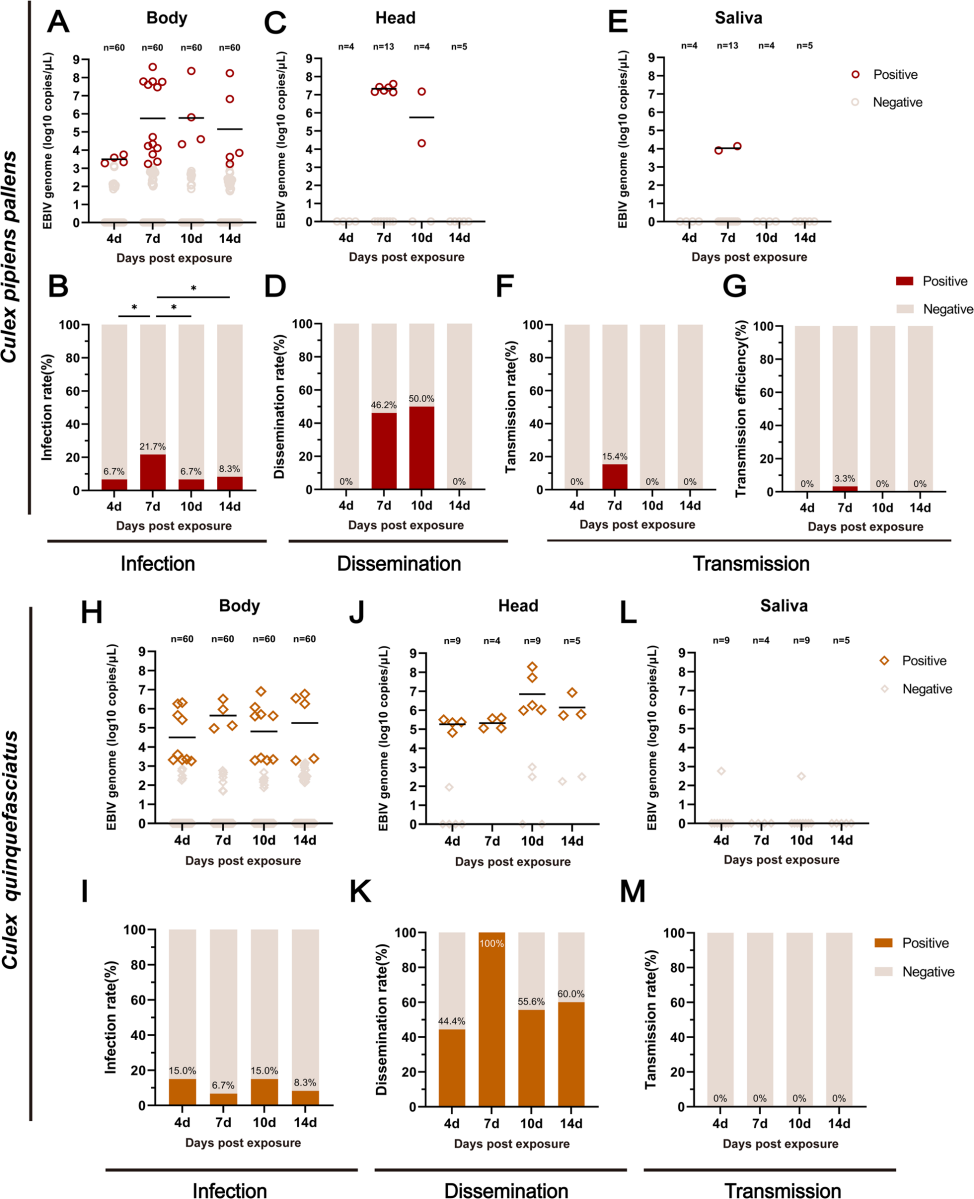
EBIV infection, dissemination, and transmission in *Culex pipiens pallens* and *Cx. quinquefasciatus*. Viral RNA copies in the bodies (**A**), heads (**C**), and saliva (**E**) samples and infection rates (**B**), dissemination rates (**D**), transmission rates (**F**), and transmission efficiency (**G**) of *Cx. pipiens pallens* at 4, 7, 10, and 14 days after feeding on a blood meal containing 3.8 × 10^6^ PFU/ml EBIV. Viral RNA copies in the body (**H**), head (**J**), and saliva (**L**) samples and infection rates (**I**), dissemination rates (**K**), and transmission rates (**M**) of *Cx. quinquefasciatus* at 4, 7, 10, and 14 days after feeding on a blood meal containing 5.7 × 10^6^ PFU/ml EBIV. Each dot represents an individual mosquito, and the gray dots indicate samples with a Ct value > 35. The infection rates were analyzed with Fisher’s exact test, and mean viral RNA copies/μl were analyzed with one-way ANOVA with Tukey’s multiple comparison (*P ≤ 0.05, **P ≤ 0.01, ***P ≤ 0.005, ****P ≤ 0.0001)

Both *Cx. pipiens pallens* and *Cx. quinquefasciatus* were used to test the EBIV dissemination and transmission. For *Cx. pipiens pallens*, six body samples were collected at 7 dpe with the viral RNA copies exceeding 10^7^ copies/μl, with a few individuals reaching ≥ 10^8^ copies/μl. Ebinur Lake virus RNA was only detected in the heads at 7 (mean value as 10^7.3^ copies/μl) and 10 dpe (mean value 10^5.7^ copies/μl), respectively, corresponding to DRs of 46.2% and 5/0.0% (Fig. 4C, D). Ebinur Lake virus RNA and infectious virus were detected in the saliva of two positive mosquitoes only at 7 dpe, with mean viral RNA copies of 10^4.0^ copies/μl (equivalent to an EBIV titer of 10^1.8^ PFU/ml) (Fig. 4E, F; Additional file 3: Fig. S1B and C) and a TR of 15.4%. Since only limited positive samples for body and saliva were detected in *Cx. pipiens pallens*, transmission efficiency indicating the potential transmission for the population was evaluated, and as shown in Fig. 4G, only 3.3% transmission efficiency was observed in *Cx. pipiens pallens*.

In addition, the IR and viral load (Fig. 4H, I) in positive *Cx. quinquefasciatus* mosquitoes exposed to EBIV were consistent with the findings of the above steps (Fig. 2C, D). The viral load for a few positive mosquitoes exceeded 10^6^ copies/μl (8 mosquitoes). Ebinur Lake virus was detected in the heads of positive mosquito samples on all tested days, and the mean viral RNA copies in the heads ranged from 10^5.3^ to 10^6.9^ copies/μl, corresponding to DRs of 44.4% to 100.0% (Fig. 4J, K), indicating the virus can break through the midgut and then disseminate to the head if the virus can reach a high level of viral load in the body. However, none of the EBIV-positive saliva samples were detected in all tested dpes (Fig. 4L, M).

## Discussion

Previous research demonstrated that the viral RNA for CQV, classified within the same viral species alongside OYAV, could be detected in *Ae. aegypti*, *Cx. quinquefasciatus*, and *Culex tritaeniorhynchus* at 12 dpi through intrathoracic and artificial membrane/oral feeding routes [33]. Our findings further indicated that *Cx. pipiens pallens* had a notably higher IR to OYAV (35.7%–40.0%) compared to *Cx. quinquefasciatus*, *Ae. albopictus*, and *Ae. aegypti* (6.3%–18.3%). *Culex pipiens pallens* showed high susceptibility to OYAV, but only very few head samples (~ 1/20) and no saliva samples were detected as positive, indicating the transmission risk for OYAV (strain SZC50) through *Cx. pipiens pallens* was very limited. Arboviruses, during their invasion process, must navigate the innate immune responses of mosquitoes and overcome several barriers, including the midgut infection barrier, midgut escape barrier (MEB), salivary gland infection barrier (SGIB), and salivary gland escape barrier (SGEB) [39]. Among these barriers, MEB might be a substantial barrier to OYAV’s hemolymph circulation entry. However, factors influencing arbovirus midgut escape are complex, such as the necessity for a virus to reach a threshold level for escape and viral dose considerations [40, 41]. The role of MEB in OYAV transmission by *Cx. pipiens pallens* requires further experimental evidence.

Our team’s earlier studies documented that *Ae. aegypti* mosquitoes could be infected by EBIV, and the virus can spread to the saliva at 14 dpi with an average viral titer exceeding 6.3 PFU per mosquito [32]. In addition, > 90% of BALB/c mice succumbed to infection with low doses of EBIV (1–10 PFU), indicating a greater pathogenicity to rodents of this virus compared to other orthobunyaviruses [23]. The current research uncovered that *Cx. pipiens pallens* and *Cx. quinquefasciatus* exhibited the highest IR at 26.7% and 21.2%, respectively, in contrast to *Ae. albopictus*. Specifically, for *Cx. pipiens pallens*, the DR was found to be 46.2%, with a TR of 15.4%, and the viral RNA (10^4.0^ copies/μl) and actual infectious viruses were detected in saliva through RT-qPCR and plaque assay (Additional file 3: Fig. S1 B and C), suggesting a potential risk of EBIV transmission from *Cx. pipiens pallens* to vertebrate hosts. In *Cx. quinquefasciatus*, the DR for EBIV ranged from 44.4 to 100%, but EBIV was not detected in the saliva of positive mosquitoes. This possibility could be that the salivary gland barrier restricted the transmission of EBIV in *Cx. quinquefasciatus* [42, 43].

Vector competence can be affected by different genotypes/isolates of the virus and different mosquito species and geographic strains. For example, experimental studies of mosquito infections through the OROV of the same Simbu serogroup have shown different IR and TR of *Cx. quinquefasciatus* infected by different OROV isolates from different geographic strains. The TR of *Cx. quinquefasciatus* from Brazil was 0.0%, but the TR of *Cx. quinquefasciatus* from Florida was 0.8%–0.9% [44, 45]. In addition, the interaction of the viruses with field mosquitoes is also important. The EBIV (Cu20-XJ) was isolated from field *Cx*. *modestus* [20], and the OYAV (SZC50) was isolated from field biting midge samples [18].

This study tested only laboratory-reared mosquitoes and one strain for each virus. The comprehensive vector competence, not only for laboratory-reared but also field-collected mosquito or midge species, should be uncovered. Furthermore, the mosquito/midge—virus—vertebrate transmission model should be developed to better understand the transmission cycle for these neglected arboviruses.

## Conclusions

Our findings indicate that *Cx. pipiens pallens* exhibited high susceptibility to OYAV (strain SZC50) compared to *Cx. quinquefasciatus*, *Ae. albopictus*, and *Ae. aegypti*, but did not efficiently disseminate the virus to saliva, indicating a limited transmission risk. Furthermore, both *Cx. pipiens pallens* and *Cx. quinquefasciatus* showed higher susceptibility to EBIV (strain Cu20-XJ), contrasting with *Ae. albopictus*. However, EBIV RNA and actual viruses were only detected in the saliva of *Cx. pipiens pallens*, indicating that transmission risk for EBIV by *Cx. pipiens pallens* exists.

### Supplementary Information

**Additional file 1: Table S1.** The correlation between OYAV and EBIV-induced cytopathic effects in BHK-21 cells and Ct values of virus RNA by qRT-PCR.

**Additional file 2: Table S2.** The equation for the standard curve was used to calculate the viral genome copies in each sample.

**Additional file 3: Figure S1.** The CPE (A) and plaque (B and C) observation to confirm actual virus with infectious presented in mosquito samples.

## Data Availability

The corresponding author can provide the datasets used and/or analyzed during the current investigation upon reasonable request.

## Abbreviations

CQV: Cat Que virus
dpe: Days post exposure
DR: Dissemination rate
EBIV: Ebinur Lake virus
IR: Infection rate
MEB: Midgut escape barrier
OYAV: Oya virus
PFU: Plaque-forming unit
SGIB: Salivary gland infection barrier
SGEB: Salivary gland escape barrier
TR: Transmission rate
