# Supplementary figures and images for: Evaluating the mosquito vector range for two orthobunyaviruses: Oya virus and Ebinur Lake virus
Source: Parasit Vectors. 2024 May 7;17:204. doi: 10.1186/s13071-024-06295-5 (PMC11077878; doi:10.1186/s13071-024-06295-5)

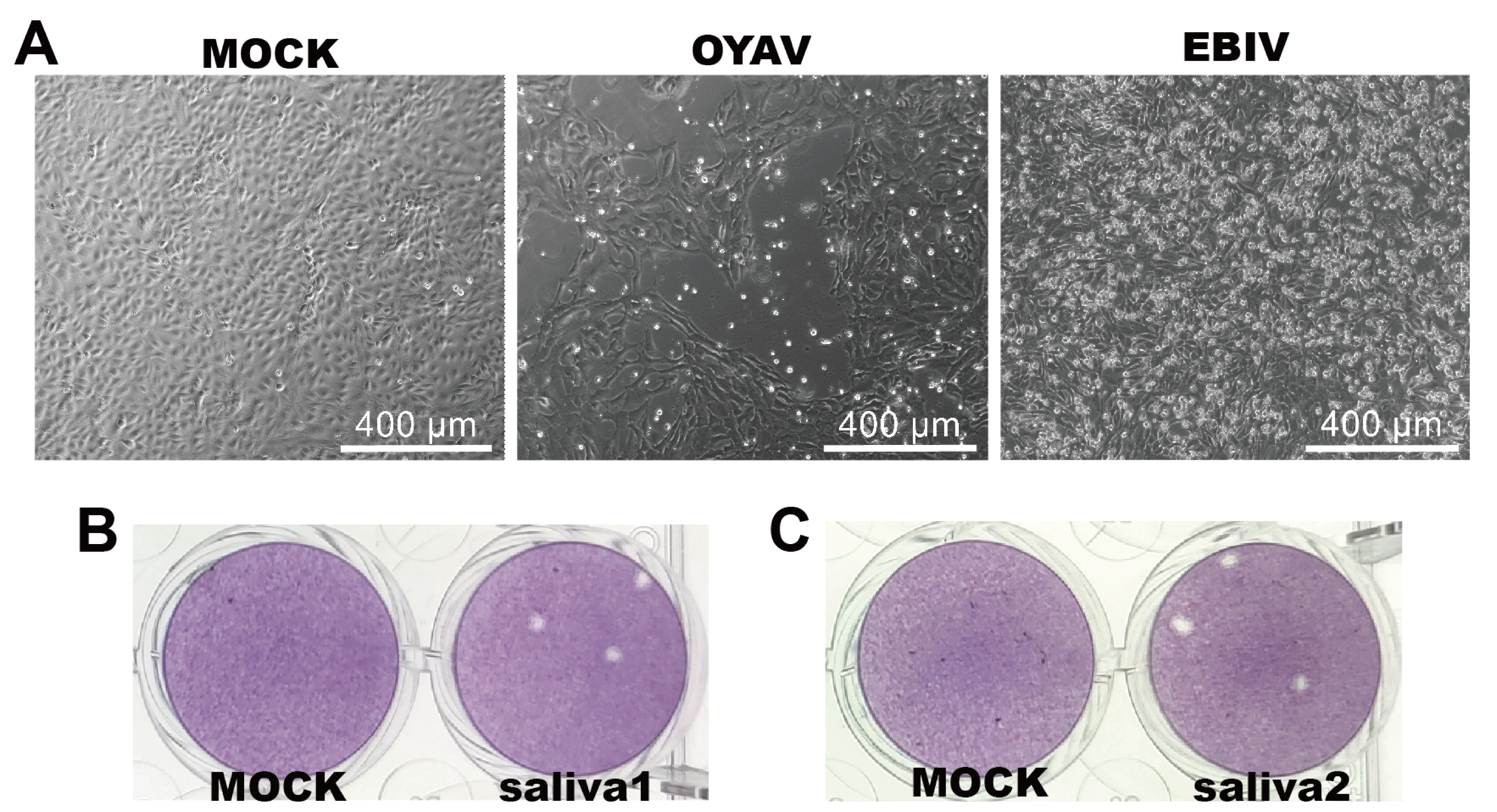

Supplement: Supplementary file 3 — Additional file 3: Figure S1. The CPE (A) and plaque (B and C) observation to confirm actual virus with infectious presented in mosquito samples. [file 13071_2024_6295_MOESM3_ESM.tif]
